# Optical coherence tomography segmentation analysis in relapsing remitting versus progressive multiple sclerosis

**DOI:** 10.1371/journal.pone.0172120

**Published:** 2017-02-13

**Authors:** Raed Behbehani, Abdullah Abu Al-Hassan, Ali Al-Salahat, Devarajan Sriraman, J. D. Oakley, Raed Alroughani

**Affiliations:** 1 Al-Bahar Ophthalmology Center, Ibn Sina Hospital, Kuwait City, Kuwait; 2 Neurology Clinic, Dasman Institute, Dasman, Kuwait; 3 National Dasman Diabetes Biobank, Dasman Institute, Dasman, Kuwait; 4 Voxeleron LLC, Pleasanton, CA, United States of America; 5 Division of Neurology, Amiri Hospital, Sharq, Kuwait; Roskamp Institute, UNITED STATES

## Abstract

**Introduction:**

Optical coherence tomography (OCT) with retinal segmentation analysis is a valuable tool in assessing axonal loss and neuro-degeneration in multiple sclerosis (MS) by in-vivo imaging, delineation and quantification of retinal layers. There is evidence of deep retinal involvement in MS beyond the inner retinal layers. The ultra-structural retinal changes in MS in different MS phenotypes can reflect differences in the pathophysiologic mechanisms. There is limited data on the pattern of deeper retinal layer involvement in progressive MS (PMS) versus relapsing remitting MS (RRMS). We have compared the OCT segmentation analysis in patients with relapsing-remitting MS and progressive MS.

**Methods:**

Cross-sectional study of 113 MS patients (226 eyes) (29 PMS, 84 RRMS) and 38 healthy controls (72 eyes). Spectral domain OCT (SDOCT) using the macular cube acquisition protocol (Cirrus HDOCT 5000; Carl Zeiss Meditec) and segmentation of the retinal layers for quantifying the thicknesses of the retinal layers. Segmentation of the retinal layers was carried out utilizing Orion software (Voxeleron, USA) for quantifying the thicknesses of individual retinal layers.

**Results:**

The retinal nerve finer layer (RNFL) (*p* = 0.023), the ganglion-cell/inner plexiform layer (GCIPL) (*p* = 0.006) and the outer plexiform layer (OPL) (*p* = 0.033) were significantly thinner in PMS compared to RRMS. There was significant negative correlation between the outer nuclear layer (ONL) and EDSS (*r* = -0.554, *p* = 0.02) in PMS patients. In RRMS patients with prior optic neuritis, the GCIPL correlated negatively (*r* = -0.317; *p* = 0.046), while the photoreceptor layer (PR) correlated positively with EDSS (*r* = 0.478; *p* = 0.003).

**Conclusions:**

Patients with PMS exhibit more atrophy of both the inner and outer retinal layers than RRMS. The ONL in PMS and the GCIPL and PR in RRMS can serve as potential surrogate of disease burden and progression (EDSS). The specific retinal layer predilection and its correlation with disability may reflect different pathophysiologic mechanisms and various stages of progression in MS.

## Introduction

Multiple sclerosis (MS) is an autoimmune demyelinating disease of the central nervous system characterized by axonal loss and neurodegeneration. Most patients with MS exhibit a relapsing remitting course initially and later on transition into a secondary progressive stage. Less commonly patients exhibit a primary progressive course from the disease onset. [1] Optical Coherence tomography (OCT) has emerged as an excellent non-invasive tool to quantitatively assess axonal loss and neurodegeneration by imaging the retina. Numerous studies were performed using spectral domain OCT (SDOCT) on relapsing remitting MS (RRMS) patients, but only few attempted to compare pattern of neuroaxonal loss and neurodegneration with progressive MS (PMS) patients, both primary (PPMS) and secondary progressive (SPMS). [2–7] Studies using time-domain OCT have attempted to explore the difference between MS subtypes based on the pattern and extent of retinal nerve fiber layer (RNFL) involvement. [4, 6, 8, 9] However, post-mortem studies have shown that patients with progressive MS have atrophy beyond the RNFL in the deeper retinal layers. [10] The development of new software algorithms using spectral-domain OCT (SDOCT) allows segmentation and thickness measurement of individual retinal layers in order to assess the effects of MS subtype on retinal layers beyond the RNFL. The primary objective of this study is to compare the OCT segmentation analysis findings among patients with RRMS and PMS subtypes. The secondary objective was to assess the correlation between thickness of individual retinal layers with neurologic disability expressed by expanded disability study scale (EDSS) scores and contrast sensitivity.

## Methods

This is a cross-sectional study conducted at the MS clinic in Dasman Institute. The diagnosis of MS was based on the revised 2010 McDonald diagnostic criteria, which requires dissemination in time and place; either clinically two relapses in two different anatomical locations or by clinical presentation supported by MRI features (dissemination in space through the presence of demyelinating plaques in different topographical locations and at different times using either gadolinium-enhancement of asymptomatic plaques or through a follow-up MRI scan showing a new T2 or Gadolinium-enhancing lesions). [11, 12]

Multiple sclerosis is classified as relapsing-remitting (RRMS) or progressive (PMS) in which there is accumulation of neurological deficients sustained over time without clear relapses. We have classified the MS patients in our cohort based on clinical presentation, i,e. RRMS or PMS subtypes. RRMS patients had a clear history of relapses (either new or recurrent) while the PMS group had a progression of neurological disability measured by mean of expanded disability status scale (EDSS). Progression is defined as an increase of at least 1-step point on EDSS scale if the patients had baseline EDSS is 5.5 or less. If the baseline EDSS score is above 5.5, half-step point on EDSS is needed to define progression. The progression has to be sustained and confirmed at a follow-up neurological examination 6 months later. The PMS group included primarily patients with both secondary progressive MS (SPMS) and few with primary progressive MS (PPMS). We have excluded patients with other disease of the retina and optic nerve, other demyelinating disorders (neuromyelitis optica or acute disseminated encephalomyelitis), and subjects with high refractive error (+- 6 diopters). [12] Patients with a recent history of optic neuritis (less than 6 months) were also excluded from the study. The occurrence of optic neuritis was verified by reviewing the medical records, direct patient questioning and/or the presence of asymmetric RNFL of more the 20% between the two eyes. All patients had neuro-ophthalmologic assessment including Snellen visual acuity and contrast sensitivity scoring using Pelli-Robson charts at one meter. Patient demographics (age, gender), clinical characteristics (age at disease onset, disease duration, presentation at onset, baseline EDSS score) were obtained from their medical records. This study was approved by the research ethics committee of Dasman Institute and the study was in accordance with the declaration of Helsinki for biomedical research. All patients provided written informed consent.

### OCT

**Fig 1:**
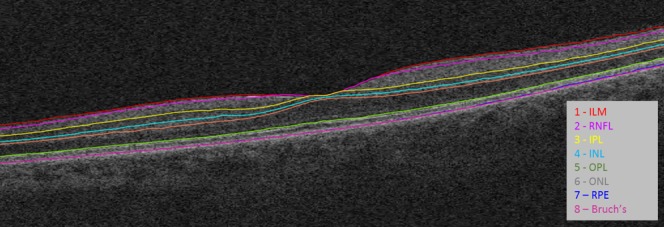
Example OCT macular scan and segmentation by the Orion^TM^ software (Voxeleron, CA). We define the layers as follows: the retinal nerve fiber layer (RNFL) as between the 1^st^ (red) and 2^nd^ segmentation lines (magenta); the ganglion cell plus inner plexiform layer complex (GC-IPL) as between the 2^nd^ (magenta) and 3^rd^ (yellow) lines; the inner nuclear layer (INL) as between the 3^rd^ (yellow) and 4^th^ (cyan) lines; the outer plexiform layer (OPL) as between the 4^th^ (cyan) and 5^th^ (orange) lines; the outer nuclear layer (ONL) as between the 5^th^ (orange) and 6^th^ (green–denoting the ellipsoid zone) lines; and the photoreceptor complex between the ellipsoid zone and the 7^th^ line, the retinal pigment epithelium layer RPE (in blue).

Subjects were scanned using SDOCT (Cirrus HDOCT 5000; Carl Zeiss Meditec) using the macular cube acquisition protocol (512x128 A-scans). Segmentation of the retinal layers was carried out utilizing Orion software (Voxeleron, USA) for quantifying the thicknesses of the macular retinal nerve fiber layer (RNFL), ganglion cell layer/inner plexiform layer (GCIPL), inner nuclear layer (INL), outer plexiform layer (OPL), outer nuclear layer (ONL) and the photoreceptor layer (PR). (Fig 1) These layers were studied in all segments of the macular ETDRS grid. The following boundaries were identified and segmented:

1. The inner limiting membrane (ILM)
2. The posterior of the retinal nerve fiber layer (RNFL). We thereby define the RNFL thickness to be the distance between the RNFL and the ILM.
3. The posterior of the inner plexiform layer (IPL). We thereby define a ganglion cell complex as the thickness between the posterior of the RNFL and the posterior of the IPL. This is designated as the GCIPL thickness.
4. The posterior of the inner nuclear layer (INL). We thereby define the INL thickness as that between the posterior of the INL and the posterior of the IPL.
5. The posterior of the outer plexiform layer (OPL). We thereby define the OPL thickness as that between the posterior of the IPL and the posterior of the INL.
6. The next boundary segmented is the ellipsoid zone (EZ) [13], which was previously referred as the photoreceptor inner segment/outer segment (IS/OS) junction. We thereby define the outer nuclear layer (ONL) thickness to be from this boundary to the posterior of the OPL. It is therefore a complex that includes the nuclei of the ONL and also the clusters of junctional complexes between the Muller cells and photoreceptors that extend from the external limiting membrane (ELM) to the IZ.
7. The last segmented boundary is the retinal pigment epithelial (RPE) band. We define the photo receptor (PR) thickness as a complex taken from this band to the EZ.

OCT scans were performed by an experienced operator and all scans were reviewed by the first author and were determined to be of good quality and high signal strength (above 6). Scans of poor quality and low signal strength (less than 6) were excluded. Pupillary dilatation was performed in patients with small pupils and whenever a poor signal strength after scanning was obtained but it was not required for all patients. Average layer thicknesses were reported within an annulus centered at the fovea by the software. The annulus covered a 6mm diameter circle, with the central 1mm circle excluded. Scans were manually reviewed for segmentation and positioning errors.

### Statistical analysis

Data were analyzed using SPSS software (ver. 23.0 for Windows; IBM SPSS Inc., Chicago, IL, USA). Descriptive statistics were calculated for all variables. Spearman rank correlation was used to determine the correlations between Progressive MS (PMS) and relapsing remitting MS (RRMS) with mean EDSS scores, disease duration and contrast sensitivity. Multiple linear regression was used to correlate with baseline EDSS score, disease duration and contrast sensitivity adjusted for age and gender to assess the retinal layer thicknesses. Coefficient of variation for inter-visit OCT measurement percentage was calculated using the standard deviation divided by the mean retinal layer thickness. *P*<0.05 was considered to be statistically significant.

## Results

A total of 113 MS (226 eyes) patients were compared to 38 healthy (72 eyes) controls. Table 1 shows the demographic and clinical characteristics of the MS patients and healthy controls. Twenty-nine (26.6%) of the MS patients had progressive MS (PMS) (Mean ± SD; 40.57 ± 8.09; range 26–56 years), while 84 (73.4%) had relapsing-remitting MS (RRMS) (Mean ± SD; 32.43 ± 9.21, range 17–61 years). There was a significant age difference observed between PMS and RRMS (p<0.001). Of the 113 MS patients 43, 38% (PMS = 6, and RRMS = 37) had prior history of optic neuritis (ON). Patients with PMS had a longer mean disease duration compared to RRMS patients (12.89 ± 6.20 vs. 6.21 ± 4.12 years; *p* < 0.001). The EDSS score was significantly higher in PMS 5.88 ± 1.49 compared to RRMS 1.78 ± 1.06 (*p* < 0.001).

**Table 1:** Clinical Characteristics and Demographics.

	PMS	RRMS	Healthy	P value
Subjects, n	29	84	38	
Primary Progressive n(%)	2 (6.9%)	-		
Secondary Progressing, n(%)	27 (93.1%)	-	-	
Age in years (Mean ± SD)	40.57 ± 8.09	32.43 ± 9.21	31.63 ± 5.74	• PMS vs. RRMS <0.001 • PMS vs. Healthy <0.001 • RRMS vs. Healthy 0.569
Range (in years)	26–56	17–61	26–51	
Gender: Male n (%) Female n (%)	• 13 (44.8%) • 16 (55.2%)	• 29 (34.5%) • 55 (65.5%)	• 13 (36.1%) • 23 (63.9%)	0.613
Baseline EDSS (Mean ± SD)	5.88 ± 1.49	1.78 ± 1.06	-	<0.001
Disease Duration (Mean ± SD) Years	12.89 ± 6.20	6.21 ± 4.12	-	<0.001
Contrast Sensitivity Right eye (Mean ± SD)	1.43 ± 0.48	1.71 ± 0.31	-	0.032
Contrast Sensitivity Left eye (Mean ± SD)	1.39 ± 0.46	1.69 ± 0.30	-	0.016
Contrast Sensitivity Binocular (Mean ± SD)	1.67 ± 0.29	3.61 ± 16.16	-	0.273
• History of ON • No history of ON	• 6 (20.7%) • 23 (79.3%)	• 37 (44.0%) • 47 (56.0%)		0.026

PMS = progressive multiple sclerosis.

RRMS = relapsing remitting multiple sclerosis.

SD = standard deviation.

EDSS = expanded disability status scale.

ON = optic neuritis.

There was significant thinning in RNFL and GCIPL and INL in both PMS and RRMS compared to healthy controls. (*p* < 0.001). The ONL was significantly thinner in RRMS compared to healthy controls (*p* = 0.001), while there was a trend towards the ONL being thinner in PMS than healthy controls (*p* = 0.056). The OPL in PMS was thinner than RRMS (*p* = 0.033) and healthy controls (p = 0.004) while the PR was significantly thicker in PMS compared to healthy controls. (*p* = 0.016) (Table 2)

**Table 2:** Mean Comparison of Retinal Layer Annulus between PMS, RRMS and Healthy Controls.

Retinal Layer	Mean ± SD			P VALUE		
	PMS; Mean ± SD	RRMS; Mean ± SD	Healthy; Mean ± SD	PMS vs. RRMS	PMS vs. Healthy	RRMS vs. Healthy
RNFL	38.63 ± 6.55	42.05 ±7.26	46.29 ±2.60	0.023	<0.001	<0.001
GCIPL	60.01 ±8.55	65.31 ±8.87	71.72± 3.15	0.006	<0.001	<0.001
INL	34.86 ± 3.52	35.48 ±2.36	37.22 ±1.45	0.387	0.001	<0.001
OPL	17.28 ±1.34	17.96 ± 1.70	18.51 ± 1.30	0.033	0.004	0.054
ONL	77.95 ± 4.18	77.14 ±5.53	80.11 ±4.11	0.416	0.056	0.001
PR	38.52 ± 2.63	37.37 ± 3.39	37.18 ± 3.00	0.066	0.016	0.759

RNFL = retinal nerve fiber layer, GCIPL = ganglion cell/inner plexiform layer, INL = inner nuclear layer, ONL = outer nuclear layer, OPL = outer plexifrom layer, PR = photoreceptor layer.

SD = standard deviation.

PMS = progressive multiple sclerosis.

RRMS = relapsing remitting multiple sclerosis.

**Fig 2:**
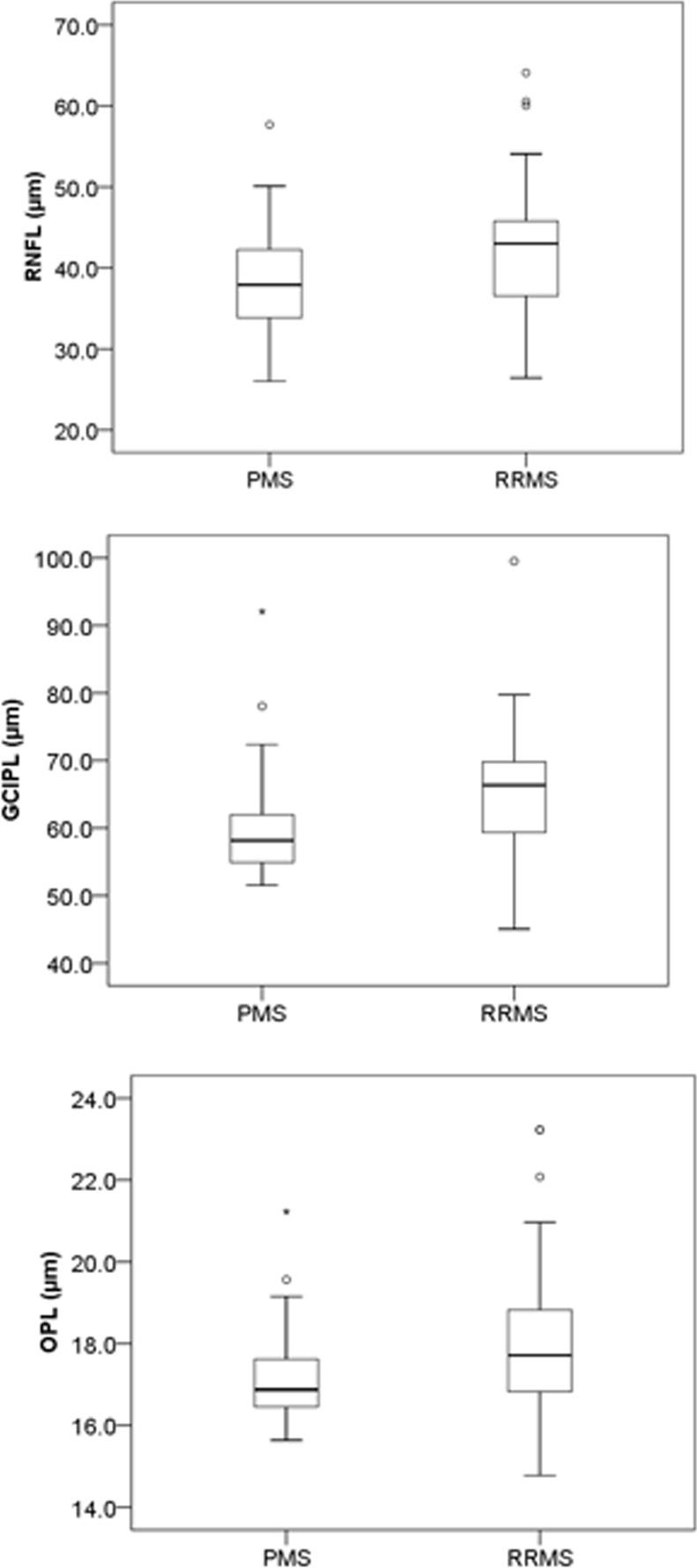
Box plot showing the significant thinning in RNFL (p = 0.023), GCIPL (p = 0.006) and OPL (p = 0.033) between PMS and RRMS. PMS = progressive multiple sclerosis; RRMS = relapsing remitting multiple sclerosis.

The RNFL (*p* = 0.023), GCIPL (*p* = 0.006) and OPL (*p* = 0.033) were significantly thinner in PMS compared to RRMS. (Table 2, Fig 2).

**Fig 3:**
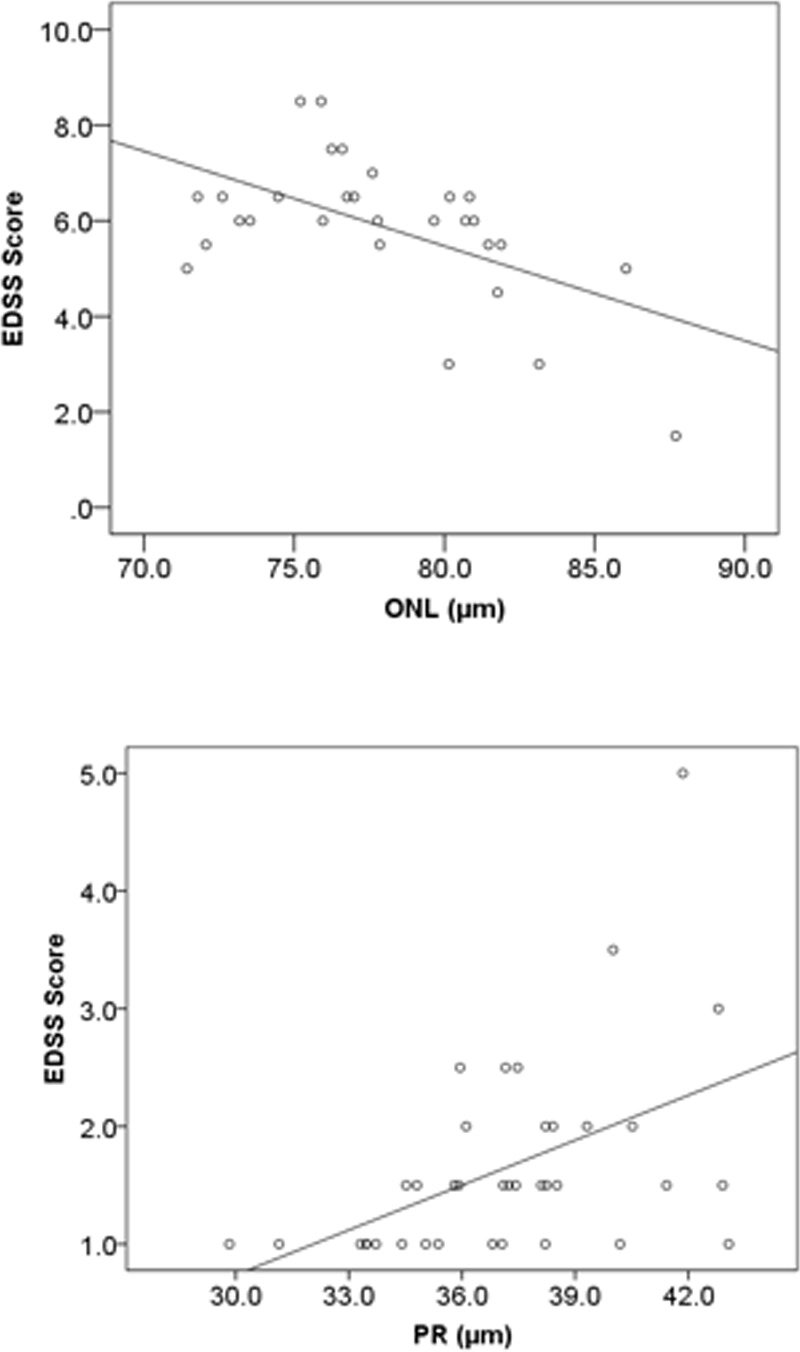
Linear regression between EDSS score in PMS and RRMS with ON. (A) Significant (r = -0.55; p = 0.002) association between EDSS score and ONL among PMS patients. (B) Significant (r = 0.48; p = 0.003) association between EDSS score and PR in RRMS prior ON.

**Fig 4:**
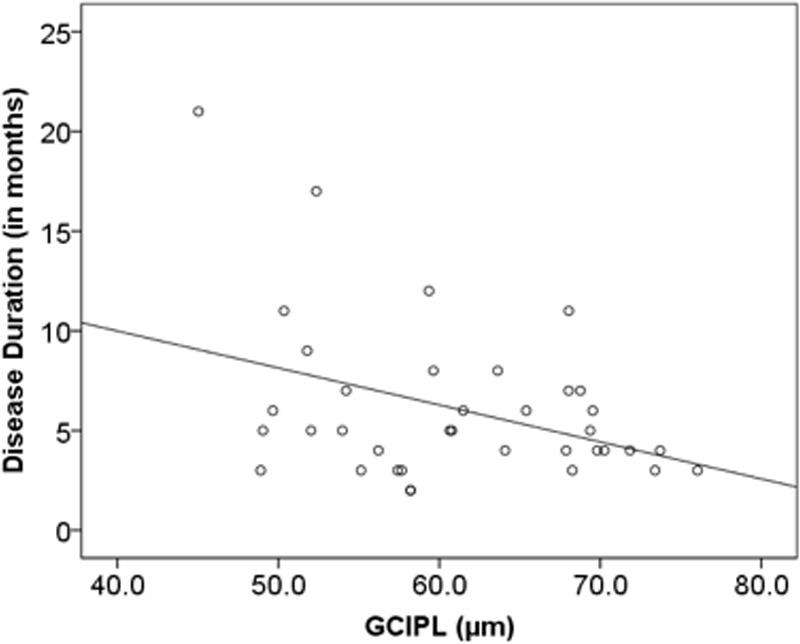
Linear regression adjusted for age (r = -0.38; p = 0.019) in RRMS patients with ON showed significant association between disease duration and annulus of GCIPL.

Multiple linear regression analysis adjusted for age was carried out to evaluate whether RNFL, GCIPIL, OPL, INL, ONL, TRT and PR were predictive of EDSS score in PMS and RRMS with ON. There was no significant correlation between any of the retinal layers with EDSS, disease duration and contrast sensitivity among RRMS subjects as a whole. However, both the RNFL (r = 0.471; *p* = 0.003) and GCIPL (r = 0.333; *p* = 0.044) correlated positively with contrast sensitivity in RRMS patients with prior history of ON. In addition, there was significant negative correlation between GCIPL and EDSS in RRMS patients with prior ON (r = -0.317; *p* = 0.046). (Table 3). We found a moderate-to-strong linear correlation (r = -0.55; p = 0.002) between EDSS score and ONL in PMS patients (Table 4, Fig 3A). In RRMS patients with prior ON, EDSS score correlated positively with PR (r = 0.48; p = 0.003) and negatively with GCIPL (-0.317, p = 0.046) (Teble3, Fig 3B). In RRMS with prior ON, disease duration correlated negatively with GCIPL (r = -0.31; p = 0.019). (Table 3, Fig 4) Finally, when adjusting for age, none of the retinal layers significantly correlated with contrast sensitivity. (Table 4)

**Table 3:** Correlation of Retinal Layer In RRMS Subjects With Mean EDSS, Disease Duration and Contrast Sensitivity.

	EDSS		DISEASE DURATION		CONTAST SENSITIVITY	
	r	P value	r	P value	r	P value
RNFL	-0.197	0.243	-0.206	0.221	0.471	0.003
GCIPL	-0.317	0.046	-0.248	0.139	0.333	0.044
INL	-0.089	0.599	-0.284	0.089	0.214	0.203
OPL	0.181	0.284	0.110	0.519	0.282	0.091
ONL	0.155	0.361	-0.218	0.195	0.170	0.316
PR	0.523	0.001	0.170	0.313	0.226	0.178

RNFL = retinal nerve fiber layer, GCIPL = ganglion cell/inner plexiform layer, INL = inner nuclear layer, ONL = outer nuclear layer, OPL = outer plexifrom layer, PR = photoreceptor layer.

EDSS = expanded disability status scale.

RRMS = relapsing-remitting multiple sclerosis.

r = spearman correlation coefficient.

ON = Optic Neuritis.

**Table 4:** Correlation of retinal layer in PMS subjects with mean EDSS, disease duration and contrast sensitivity.

	EDSS		DISEASE DURATION		CONTAST SENSITIVITY	
	r	P value	r	P value	r	P value
RNFL	-.108	.577	-.078	.694	.186	.491
GCIPL	-.050	.796	.038	.849	.230	.391
INL	-.173	.370	-.113	.567	.055	.840
OPL	-.177	.357	.091	.646	.306	.249
ONL	-.554	**.002**	-.145	.461	.037	.892
PR	.056	.772	.242	.216	-.433	.094

RNFL = retinal nerve fiber layer, GCIPL = ganglion cell/inner plexiform layer, INL = inner nuclear layer, ONL = outer nuclear layer, OPL = outer plexifrom layer, PR = photoreceptor layer.

EDSS = expanded disability status scale.

PMS = Progressive multiple sclerosis.

r = spearman correlation coefficient.

The RNFL and GCIPL had the highest coefficient of variation (COV) in both PMS and RRMS patients; while the COV was generally low in all retinal layers in healthy controls. (Table 5)

**Table 5:** Coefficient of variation (COV%) in PMS, RRMS, and Healthy Control Subjects.

Retinal Layer Annulus	PMS	RRMS	Healthy
RNFL	16.96	17.26	5.61
GCIPL	14.25	13.58	4.39
INL	10.10	6.65	3.89
ONL	8.52	9.34	5.89
OPL	7.75	9.45	7.00
PR	6.82	9.09	8.07

RNFL = retinal nerve fiber layer, GCIPL = ganglion cell/inner plexiform layer, INL = inner nuclear layer, ONL = outer nuclear layer, OPL = outer plexiform layer, PR = photoreceptor layer.

PMS = Progressive multiple sclerosis, RRMS = relapsing-remitting multiple sclerosis.

## Discussion

We have found significant RNFL and GCIPL thinning in PMS compared to RRMS patients. This indicates that neuro-degeneration manifested by axonal and ganglion cell loss is accelerated in patients with more progressive phases of MS independent of disease duration, age and history of prior optic neuritis. Furthermore, GCIPL thinning in other studies was found to be associated with disease progression, new T-2 lesions and Godalinium-enhancing lesions. [6, 14] Post-mortem histopathological studies have demonstrated evidence of retinal inflammation and periphlebitis in the deep retinal layers due to blood-retinal barrier disruption. [10, 15] We have also found that GCIPL was strongly correlated with EDSS and visual function represented by contrast sensitivity in patients with RRMS and prior ON, while the RNFL did not (Table 3). This suggests that in our cohort of RRMS patients with prior ON, GCIPL is a better index to assess disease severity and progression and visual function than RNFL. In another study, the RNFL was found to correlate better with EDSS in patients with no prior ON. [16]

The INL was significantly thinner in in both patients with RRMS and PMS compared to controls. The ONL was significantly thinner in RRMS while there was a trend in PMS compared to healthy controls. (Table 2) Post-mortem histopathological studies showed that atrophy of the INL can occur in up to 40% of cases of MS and this was thought to be due to trans-synaptic degeneration rather than a primary retinal process. [10] In our PMS cohort, OPL was significantly thinner in PMS compared to RRMS and healthy controls. (Table 2) Evidence of outer retinal involvement in MS is derived from the detection of multifocal electro-retinographic abnormalities in MS patients with unique symptoms such as glare and photophobia, which are not typical of optic neuropathy [17, 18] Recent longitudinal studies assessing deeper retinal layer changes following acute optic neuritis have shown that the INL and ONL increase in thickness in the period between 4–12 months following optic neuritis with reversion to baseline values at latest follow up. [19] Finally, other cross-sectional studies using retinal segmentation in the paramacular area of patients with RRMS have shown that optic neuritis had no effect on the outer retinal layers including the OPL, ONL, and PR layer and the effect was limited to the inner retinal layers (RNFL, GCIPL, INL). [20] This raises the possibility of other mechanisms independent of retrograde trans-synaptic degeneration following optic neuritis to be responsible of the underlying alterations in the deeper retinal layers.

Saidha et al. described a subset of RRMS patients with “predominant macular thinning” characterized by significant thinning of both the INL and ONL and relative sparing of the ganglion cell layer. Those patients had more rapid disability progression and a subset of them had multi-focal electroretinographic abnormalities. This was hypothesized to be due to a primary retinal pathology which is surrogate for more aggressive disease. [17] We have found significant thickening of the PR layer in PMS patients compared to healthy controls (0.016) and a trend towards thickening as well compared to RRMS patients (0.066), while the PR layer was not significantly different in RRMS compared to healthy controls. In the “predominant macular thinning” phenotyoe reported by Saidha et al., measurement of the INL included the PR layer as well as one complex. This was probably based on the assumption that the photoreceptor complex would be relatively constant with disease. With improvements in the analysis software, we were able to delineate these layers and have found significant thickening of the PR layer in compared to healthy controls. (Table 2) Moreover, using linear regression we found that the PR layer positively correlated with EDSS score in RRMS patients with prior history of ON (*r* = 0.523, *p* = 0.001). Using multiple regression analysis and by including GCIPL as a covariant, this remained statistically significant and therefore the thickening of PR is not simply “compensatory” thickening of the PR secondary to pronounced GCIPL thinning. To our knowledge, this finding has not been previously reported before. Nevertheless, longitudinal studies are needed to determine if the thickening of the PR layer is sustained or transient in nature.

We have found significant negative correlation between the ONL and EDSS in our PMS cohort. (Table 4) This suggests that the ONL may serve as a potential index of neurological disability and disease progression in PMS patients. Albrecht et al. have found positive correlation between the OPL and EDSS, while we did not find such correlation in our cohort. They also found significant thinning of the INL in PPMS compared to RRMS, which suggests retinal pathology involving the INL in PPMS. [4] Other studies, however, have found that increased INL thickness correlates with worsened brain atrophy specifically in PMS and predictive of disease activity (non-ocular relapse, new T2 and contrast-enhancing lesions) and disease progression. [5, 21]. This discrepancy between studies can be possibly explained by a dynamic nature of the INL thickness in MS disease course. The mechanism behind this can be retinal inflammation leading to glial cell death with impairment of their ability to process interstitial edema leading to hypertrophy of the cell bodies in the INL. Longitudinal studies are required to fully clarify the natural course of the alterations of deeper retinal layer in MS and how they correlate with disease activity and progressive disability. [22]

Our study is limited by its cross-sectional design and its relatively small sample size. Most of our PMS cohort were composed of SPMS with underrepresentation of PPMS due to rarity of the latter phenotype. In addition, we have combined the primary and secondary progressive cohort as a single group, which may have influenced our findings. However, there is increasing evidence of the phenotypic similarities between PPMS and SPMS and, common genetic susceptibility to MS are similar between and measures of global brain tissue damage and magnetization transfer imaging. [23, 24] Despite that, the OCT findings in our study in the progressive cohort do not strictly apply to PPMS, which does have its unique aspects of indolent course with less frequent visual pathway involvement and thus relative preservation of the inner retinal layers. [9]

In summary, we have significant thinning in the RNFL, GCIPL and OPL in PMS patients compared to RRMS. In PMS patients, the ONL was negatively correlated with EDSS, while in patients with RRMS and prior ON, the GCIPL correlated negatively and the PR layers correlated positively with EDSS. This has may have implications in clinical assessment of progression in MS and setting outcome measure in therapeutic trials. This predilection for specific retinal layer involvement and its correlation with disease progression may reflect differences in the pathophysiologic mechanisms and the various stages of progression MS. Longitudinal studies using OCT segmentation analysis can better define the significance and the dynamics of the changes in retinal layers in different MS phenotypes and how they relate to disease progression.

## References

[1] Tremlett H, Zhao Y, Rieckmann P, Hutchinson M. New perspectives in the natural history of multiple sclerosis. Neurology. 2010;74(24):2004–15. 10.1212/WNL.0b013e3181e3973f 20548045

[2] Serbecic N, Aboul-Enein F, Beutelspacher SC, Graf M, Kircher K, Geitzenauer W, et al Heterogeneous pattern of retinal nerve fiber layer in multiple sclerosis. High resolution optical coherence tomography: potential and limitations. PLoS One. 2010;5(11):e13877 PubMed Central PMCID: PMCPMC2975633. 10.1371/journal.pone.0013877 21079732 PMC2975633

[3] Saidha S, Syc SB, Durbin MK, Eckstein C, Oakley JD, Meyer SA, et al Visual dysfunction in multiple sclerosis correlates better with optical coherence tomography derived estimates of macular ganglion cell layer thickness than peripapillary retinal nerve fiber layer thickness. Mult Scler. 2011;17(12):1449–63. 10.1177/1352458511418630 21865411

[4] Albrecht P, Ringelstein M, Muller AK, Keser N, Dietlein T, Lappas A, et al Degeneration of retinal layers in multiple sclerosis subtypes quantified by optical coherence tomography. Multiple sclerosis. 2012;18(10):1422–9. 10.1177/1352458512439237 22389411

[5] Oberwahrenbrock T, Schippling S, Ringelstein M, Kaufhold F, Zimmermann H, Keser N, et al Retinal damage in multiple sclerosis disease subtypes measured by high-resolution optical coherence tomography. Mult Scler Int. 2012;2012:530305 PubMed Central PMCID: PMCPMC3410317. 10.1155/2012/530305 22888431 PMC3410317

[6] Balk L, Tewarie P, Killestein J, Polman C, Uitdehaag B, Petzold A. Disease course heterogeneity and OCT in multiple sclerosis. Multiple sclerosis. 2014;20(9):1198–206. 10.1177/1352458513518626 24402036

[7] Serbecic N, Aboul-Enein F, Beutelspacher SC, Khan A, Vass C, Kristoferitsch W, et al High-Resolution Spectral Domain-Optical Coherence Tomography in Multiple Sclerosis, Part II—the Total Macular Volume. The First Follow-Up Study over 2 Years. Frontiers in neurology. 2014;5:20 PubMed Central PMCID: PMCPMC3932446. 10.3389/fneur.2014.00020 24605107 PMC3932446

[8] Pulicken M, Gordon-Lipkin E, Balcer LJ, Frohman E, Cutter G, Calabresi PA. Optical coherence tomography and disease subtype in multiple sclerosis. Neurology. 2007;69(22):2085–92. 10.1212/01.wnl.0000294876.49861.dc 18040015

[9] Gelfand JM, Goodin DS, Boscardin WJ, Nolan R, Cuneo A, Green AJ. Retinal axonal loss begins early in the course of multiple sclerosis and is similar between progressive phenotypes. PloS one. 2012;7(5):e36847 PubMed Central PMCID: PMCPMC3359324. 10.1371/journal.pone.0036847 22666330 PMC3359324

[10] Green AJ, McQuaid S, Hauser SL, Allen IV, Lyness R. Ocular pathology in multiple sclerosis: retinal atrophy and inflammation irrespective of disease duration. Brain. 2010;133(Pt 6):1591–601. PubMed Central PMCID: PMCPMC2877904. 10.1093/brain/awq080 20410146 PMC2877904

[11] Weinshenker BG, Issa M, Baskerville J. Meta-analysis of the placebo-treated groups in clinical trials of progressive MS. Neurology. 1996;46(6):1613–9. 8649559 10.1212/wnl.46.6.1613

[12] Polman CH, Reingold SC, Banwell B, Clanet M, Cohen JA, Filippi M, et al Diagnostic criteria for multiple sclerosis: 2010 revisions to the McDonald criteria. Annals of neurology. 2011;69(2):292–302. PubMed Central PMCID: PMCPMC3084507. 10.1002/ana.22366 21387374 PMC3084507

[13] Staurenghi G, Sadda S, Chakravarthy U, Spaide RF, International Nomenclature for Optical Coherence Tomography P. Proposed lexicon for anatomic landmarks in normal posterior segment spectral-domain optical coherence tomography: the IN*OCT consensus. Ophthalmology. 2014;121(8):1572–8. 10.1016/j.ophtha.2014.02.023 24755005

[14] Ratchford JN, Saidha S, Sotirchos ES, Oh JA, Seigo MA, Eckstein C, et al Active MS is associated with accelerated retinal ganglion cell/inner plexiform layer thinning. Neurology. 2013;80(1):47–54. PubMed Central PMCID: PMCPMC3589201. 10.1212/WNL.0b013e31827b1a1c 23267030 PMC3589201

[15] Shindler KS, Ventura E, Dutt M, Rostami A. Inflammatory demyelination induces axonal injury and retinal ganglion cell apoptosis in experimental optic neuritis. Exp Eye Res. 2008;87(3):208–13. PubMed Central PMCID: PMCPMC2564281. 10.1016/j.exer.2008.05.017 18653182 PMC2564281

[16] Behbehani R, Al-Hassan AA, Al-Khars A, Sriraman D, Alroughani R. Retinal nerve fiber layer thickness and neurologic disability in relapsing-remitting multiple sclerosis. Journal of the neurological sciences. 2015;359(1–2):305–8. 10.1016/j.jns.2015.11.017 26671132

[17] Saidha S, Syc SB, Ibrahim MA, Eckstein C, Warner CV, Farrell SK, et al Primary retinal pathology in multiple sclerosis as detected by optical coherence tomography. Brain: a journal of neurology. 2011;134(Pt 2):518–33. 21252110 10.1093/brain/awq346

[18] Papakostopoulos D, Fotiou F, Hart JC, Banerji NK. The electroretinogram in multiple sclerosis and demyelinating optic neuritis. Electroencephalogr Clin Neurophysiol. 1989;74(1):1–10. 2463143 10.1016/0168-5597(89)90045-2

[19] Al-Louzi OA, Bhargava P, Newsome SD, Balcer LJ, Frohman EM, Crainiceanu C, et al Outer retinal changes following acute optic neuritis. Multiple sclerosis. 2016;22(3):362–72. PubMed Central PMCID: PMCPMC4724567. 10.1177/1352458515590646 26209589 PMC4724567

[20] Garcia-Martin E, Polo V, Larrosa JM, Marques ML, Herrero R, Martin J, et al Retinal layer segmentation in patients with multiple sclerosis using spectral domain optical coherence tomography. Ophthalmology. 2014;121(2):573–9. 10.1016/j.ophtha.2013.09.035 24268855

[21] Saidha S, Sotirchos ES, Ibrahim MA, Crainiceanu CM, Gelfand JM, Sepah YJ, et al Microcystic macular oedema, thickness of the inner nuclear layer of the retina, and disease characteristics in multiple sclerosis: a retrospective study. Lancet neurology. 2012;11(11):963–72. PubMed Central PMCID: PMCPMC3533139. 10.1016/S1474-4422(12)70213-2 23041237 PMC3533139

[22] Balk LJ, Cruz-Herranz A, Albrecht P, Arnow S, Gelfand JM, Tewarie P, et al Timing of retinal neuronal and axonal loss in MS: a longitudinal OCT study. J Neurol. 2016. 10.1007/s00415-016-8127-y PMC4929170 27142714

[23] Isobe N, Damotte V, Lo Re V, Ban M, Pappas D, Guillot-Noel L, et al Genetic burden in multiple sclerosis families. Genes Immun. 2013;14(7):434–40. PubMed Central PMCID: PMCPMC4102601. 10.1038/gene.2013.37 23903824 PMC4102601

[24] Rovaris M, Bozzali M, Santuccio G, Ghezzi A, Caputo D, Montanari E, et al In vivo assessment of the brain and cervical cord pathology of patients with primary progressive multiple sclerosis. Brain: a journal of neurology. 2001;124(Pt 12):2540–9. 11701606 10.1093/brain/124.12.2540

